# Crystal structure of 2-(3-nitro­phen­yl)-1,3-thia­zolo[4,5-*b*]pyridine

**DOI:** 10.1107/S2056989015019118

**Published:** 2015-10-24

**Authors:** Gamal A. El-Hiti, Keith Smith, Amany S. Hegazy, Mansour D. Ajarim, Benson M. Kariuki

**Affiliations:** a Cornea Research Chair, Department of Optometry, College of Applied Medical Sciences, King Saud University, PO Box 10219, Riyadh 11433, Saudi Arabia; b School of Chemistry, Cardiff University, Main Building, Park Place, Cardiff CF10 3AT, Wales; c Criminal Evidence, Ministry of Interior, Riyadh 11632, PO Box 86985, Saudi Arabia

**Keywords:** crystal structure, nitro­phen­yl, thia­zolo­pyridine derivatives, thia­zolo[4,5-*b*]pyridine

## Abstract

In the title compound, C_12_H_7_N_3_O_2_S, the dihedral angle between the planes of the thia­zolo­pyridine ring system (r.m.s. deviation = 0.005 Å) and the benzene ring is 3.94 (6)°. The nitro group is rotated by 7.6 (2)° from its attached ring. In the crystal, extensive aromatic π–π stacking [shortest centroid–centroid separation = 3.5295 (9) Å] links the mol­ecules into (001) sheets.

## Related literature   

For a related structure and background references, see: El-Hiti *et al.* (2015 ▸). For further synthetic details, see: Smith *et al.* (1995 ▸); El-Hiti (2003 ▸).
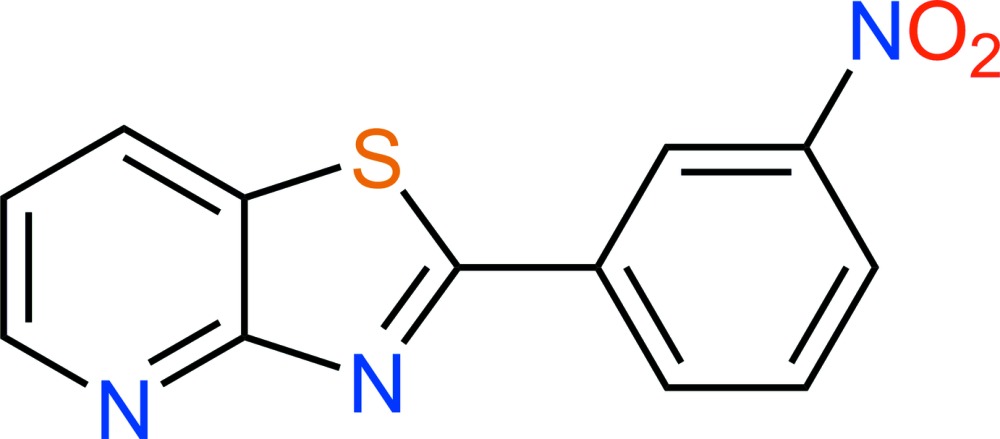


## Experimental   

### Crystal data  

C_12_H_7_N_3_O_2_S
*M* *_r_* = 257.27
Monoclinic, 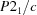

*a* = 9.5596 (2) Å
*b* = 9.8733 (2) Å
*c* = 11.5606 (3) Å
β = 98.122 (2)°
*V* = 1080.20 (4) Å^3^
*Z* = 4
Cu *K*α radiation
μ = 2.66 mm^−1^
*T* = 296 K
0.36 × 0.24 × 0.03 mm

### Data collection  

Agilent SuperNova Dual Source diffractometer with an Atlas detector
Absorption correction: Gaussian (*CrysAlis PRO*; Agilent, 2014 ▸) *T*_min_ = 0.883, *T*_max_ = 0.986
4063 measured reflections
2104 independent reflections
1930 reflections with *I* > 2σ(*I*)
*R*_int_ = 0.016

### Refinement  

*R*[*F* ^2^ > 2σ(*F* ^2^)] = 0.031
*wR*(*F* ^2^) = 0.086
*S* = 1.06
2104 reflections
163 parameters
H-atom parameters constrained
Δρ_max_ = 0.20 e Å^−3^
Δρ_min_ = −0.27 e Å^−3^

### 

Data collection: *CrysAlis PRO* (Agilent, 2014 ▸); cell refinement: *CrysAlis PRO*; data reduction: *CrysAlis PRO*; program(s) used to solve structure: *SHELXS97* (Sheldrick, 2008 ▸); program(s) used to refine structure: *SHELXL2013* (Sheldrick, 2015 ▸); molecular graphics: *ORTEP-3 for Windows* (Farrugia, 2012 ▸); software used to prepare material for publication: *WinGX* (Farrugia, 2012 ▸) and *CHEMDRAW Ultra* (Cambridge Soft, 2001 ▸).

## Supplementary Material

Crystal structure: contains datablock(s) I, New_Global_Publ_Block. DOI: 10.1107/S2056989015019118/hb7519sup1.cif

Structure factors: contains datablock(s) I. DOI: 10.1107/S2056989015019118/hb7519Isup2.hkl

Supporting information file. DOI: 10.1107/S2056989015019118/hb7519Isup3.cml

**Fig. 1.**
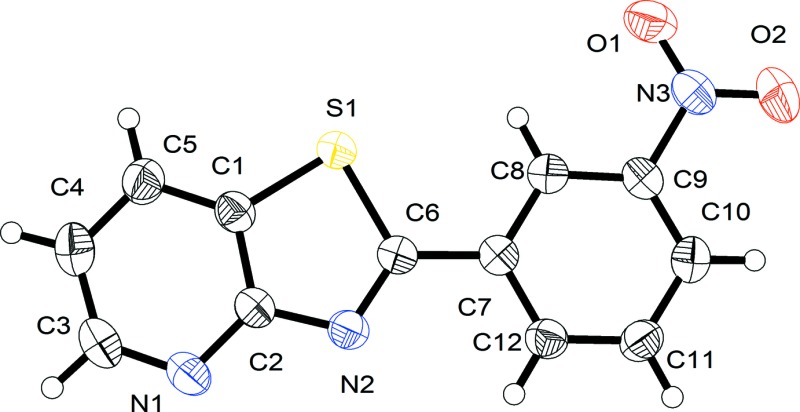
The asymmetric unit of C12H7N3O2S with 50% probability displacement ellipsoids for nonhydrogen atoms.

12 7 3 2 . DOI: 10.1107/S2056989015019118/hb7519fig1.tif
The asymmetric unit of C_12_H_7_N_3_O_2_S with 50% probability displacement ellipsoids for nonhydrogen atoms.

**Fig. 2.**
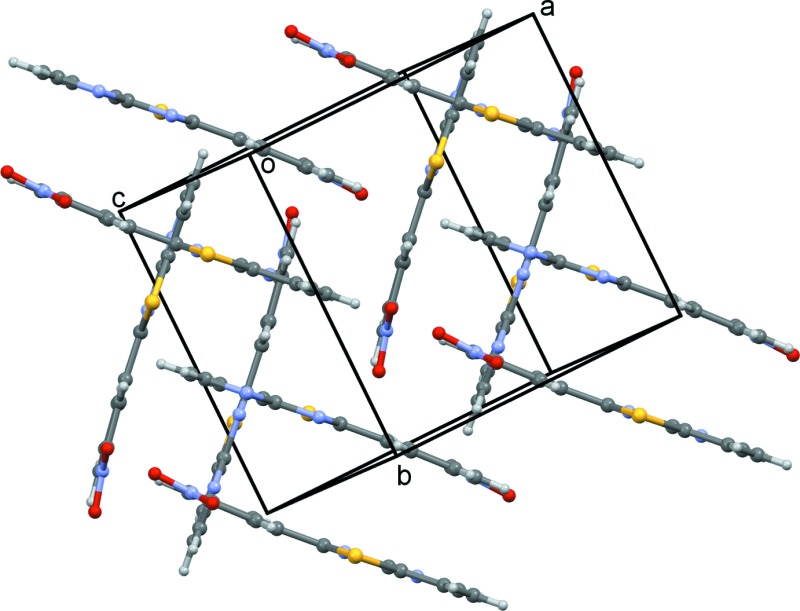
A segment of the crystal structure with H atoms omitted for clarity.

. DOI: 10.1107/S2056989015019118/hb7519fig2.tif
A segment of the crystal structure showing with H atoms omitted for clarity.

CCDC reference: 1430578

Additional supporting information:  crystallographic information; 3D view; checkCIF report

## supplementary crystallographic information

### S1. Introduction

As part of our ongoing studies of thia­zolo­pyridines (El-Hiti *et al.*, 2015), the title compound was prepared by two different processes (El-Hiti, 2003; Smith *et al.*, 1995) and its structure was determined.

### S2.1. Synthesis and crystallization

2-(3-Nitro­phenyl)-1,3-thia­zolo[4,5-*b*]pyridine was obtained in 90% yield from acid hydrolysis (HCl, 5 M) of 3-(diiso-propyl­amino­thio­carbonyl­thio)-2-(3-nitro­phenyl­carbonyl­amino)­pyridine under reflux for 5 h (Smith *et al.*, 1995) or in 58% yield from reaction of 3-(diiso­propyl­amino­thio­carbonyl­thio)-2-amino­pyridine with 3-nitro­benzoic acid in the presence of phospho­rus oxychloride under reflux for 4 h (El-Hiti, 2003). Crystallization of the crude product from chloro­form gave the title compound as colourless crystals. The structure of the title compound was elucidated by various spectroscopic and analytical data, which were consistent with those reported (Smith *et al.*, 1995).

### S2.2. Refinement

H atoms were positioned geometrically and refined using a riding model with *U*_ĩso_(H) constrained to be 1.2 times *U*_eq_ for the atom it is bonded to.

### S3. Results and discussion

The asymmetric unit comprises one molecule of C_12_H_7_N_3_O_2_S (Fig. 1). The phenyl­thia­zolo­pyridine ring system is flat with a maximum deviation of 0.072 (1)Å from the least squares plane. The nitro group is twisted from this plane by only 7.6 (2)°. In the crystal, extensive π - π overlap occurs between pairs of inversion related molecules with a phenyl to thia­zolo­pyridine centroid distance of 3.47 (2)Å (Fig. 2).

### Crystal data

**Table:**

C_12_H_7_N_3_O_2_S	*F*(000) = 528
*M**_r_* = 257.27	*D*_x_ = 1.582 Mg m^−^^3^
Monoclinic, *P*2_1_/*c*	Cu *K*α radiation, λ = 1.54184 Å
*a* = 9.5596 (2) Å	Cell parameters from 2610 reflections
*b* = 9.8733 (2) Å	θ = 5.9–73.8°
*c* = 11.5606 (3) Å	µ = 2.66 mm^−^^1^
β = 98.122 (2)°	*T* = 296 K
*V* = 1080.20 (4) Å^3^	Plate, colourless
*Z* = 4	0.36 × 0.24 × 0.03 mm

### Data collection

**Table:**

Agilent SuperNova Dual Source diffractometer with an Atlas detector	1930 reflections with *I* > 2σ(*I*)
ω scans	*R*_int_ = 0.016
Absorption correction: gaussian (*CrysAlis PRO*; Agilent, 2014)	θ_max_ = 73.8°, θ_min_ = 5.9°
*T*_min_ = 0.883, *T*_max_ = 0.986	*h* = −6→11
4063 measured reflections	*k* = −12→10
2104 independent reflections	*l* = −13→14

### Refinement

**Table:**

Refinement on *F*^2^	0 restraints
Least-squares matrix: full	Hydrogen site location: inferred from neighbouring sites
*R*[*F*^2^ > 2σ(*F*^2^)] = 0.031	H-atom parameters constrained
*wR*(*F*^2^) = 0.086	*w* = 1/[σ^2^(*F*_o_^2^) + (0.048*P*)^2^ + 0.2004*P*] where *P* = (*F*_o_^2^ + 2*F*_c_^2^)/3
*S* = 1.06	(Δ/σ)_max_ = 0.001
2104 reflections	Δρ_max_ = 0.20 e Å^−^^3^
163 parameters	Δρ_min_ = −0.27 e Å^−^^3^

### Special details

**Table:**

Experimental. Absorption correction: CrysAlisPro, Agilent Technologies, Version 1.171.37.33 (release 27-03-2014 CrysAlis171 .NET) (compiled Mar 27 2014,17:12:48) Numerical absorption correction based on gaussian integration over a multifaceted crystal model Empirical absorption correction using spherical harmonics, implemented in SCALE3 ABSPACK scaling algorithm.
Geometry. All e.s.d.'s (except the e.s.d. in the dihedral angle between two l.s. planes) are estimated using the full covariance matrix. The cell e.s.d.'s are taken into account individually in the estimation of e.s.d.'s in distances, angles and torsion angles; correlations between e.s.d.'s in cell parameters are only used when they are defined by crystal symmetry. An approximate (isotropic) treatment of cell e.s.d.'s is used for estimating e.s.d.'s involving l.s. planes.

### Fractional atomic coordinates and isotropic or equivalent isotropic displacement parameters (Å^2^)

**Table:**

	*x*	*y*	*z*	*U*_iso_*/*U*_eq_	
C1	1.03467 (14)	0.31902 (15)	0.40820 (12)	0.0359 (3)	
C2	1.05966 (14)	0.29483 (14)	0.52938 (12)	0.0346 (3)	
C3	1.23544 (16)	0.44974 (17)	0.55180 (15)	0.0466 (4)	
H3	1.3056	0.4963	0.5999	0.056*	
C4	1.21719 (17)	0.48032 (17)	0.43313 (15)	0.0476 (4)	
H4	1.2737	0.5453	0.4044	0.057*	
C5	1.11469 (16)	0.41357 (17)	0.35833 (14)	0.0446 (3)	
H5	1.1001	0.4312	0.2785	0.054*	
C6	0.88486 (14)	0.15122 (14)	0.48354 (11)	0.0327 (3)	
C7	0.78177 (13)	0.04398 (14)	0.49734 (11)	0.0323 (3)	
C8	0.69113 (14)	−0.00588 (14)	0.40184 (12)	0.0338 (3)	
H8	0.6911	0.0309	0.3278	0.041*	
C9	0.60130 (13)	−0.11128 (14)	0.41963 (12)	0.0342 (3)	
C10	0.59533 (15)	−0.16807 (15)	0.52740 (13)	0.0392 (3)	
H10	0.5333	−0.2386	0.5365	0.047*	
C11	0.68472 (16)	−0.11691 (16)	0.62203 (13)	0.0419 (3)	
H11	0.6827	−0.1532	0.6960	0.050*	
C12	0.77673 (15)	−0.01253 (16)	0.60750 (12)	0.0381 (3)	
H12	0.8362	0.0206	0.6719	0.046*	
N1	1.16011 (14)	0.35893 (14)	0.60175 (11)	0.0442 (3)	
N2	0.97356 (13)	0.19809 (13)	0.56961 (10)	0.0367 (3)	
N3	0.50993 (13)	−0.16657 (14)	0.31766 (11)	0.0420 (3)	
O1	0.51160 (13)	−0.11243 (14)	0.22310 (10)	0.0563 (3)	
O2	0.43804 (16)	−0.26586 (16)	0.33152 (13)	0.0698 (4)	
S1	0.89807 (4)	0.21799 (4)	0.34482 (3)	0.03972 (13)	

### Atomic displacement parameters (Å^2^)

**Table:**

	*U*^11^	*U*^22^	*U*^33^	*U*^12^	*U*^13^	*U*^23^
C1	0.0339 (7)	0.0341 (7)	0.0386 (7)	0.0002 (5)	0.0009 (5)	−0.0009 (6)
C2	0.0322 (7)	0.0348 (7)	0.0361 (7)	0.0004 (5)	0.0023 (5)	−0.0029 (5)
C3	0.0387 (7)	0.0429 (8)	0.0565 (9)	−0.0067 (6)	0.0004 (6)	−0.0100 (7)
C4	0.0423 (8)	0.0379 (8)	0.0627 (10)	−0.0072 (6)	0.0075 (7)	0.0012 (7)
C5	0.0445 (8)	0.0431 (8)	0.0458 (8)	−0.0041 (6)	0.0048 (6)	0.0068 (6)
C6	0.0318 (6)	0.0337 (7)	0.0322 (6)	0.0021 (5)	0.0037 (5)	−0.0016 (5)
C7	0.0293 (6)	0.0323 (6)	0.0355 (7)	0.0035 (5)	0.0049 (5)	−0.0017 (5)
C8	0.0316 (6)	0.0351 (7)	0.0346 (6)	0.0035 (5)	0.0047 (5)	−0.0004 (5)
C9	0.0279 (6)	0.0342 (7)	0.0398 (7)	0.0040 (5)	0.0024 (5)	−0.0050 (5)
C10	0.0353 (7)	0.0349 (7)	0.0479 (8)	−0.0002 (6)	0.0080 (6)	0.0028 (6)
C11	0.0436 (8)	0.0443 (8)	0.0380 (7)	0.0000 (6)	0.0060 (6)	0.0068 (6)
C12	0.0362 (7)	0.0417 (8)	0.0355 (7)	0.0001 (6)	0.0021 (5)	−0.0002 (6)
N1	0.0410 (6)	0.0476 (7)	0.0420 (7)	−0.0059 (5)	−0.0012 (5)	−0.0079 (6)
N2	0.0358 (6)	0.0401 (6)	0.0335 (6)	−0.0017 (5)	0.0029 (5)	−0.0017 (5)
N3	0.0352 (6)	0.0425 (7)	0.0467 (7)	0.0013 (5)	0.0006 (5)	−0.0082 (6)
O1	0.0582 (7)	0.0652 (8)	0.0421 (6)	−0.0030 (6)	−0.0052 (5)	−0.0054 (6)
O2	0.0684 (8)	0.0638 (9)	0.0724 (9)	−0.0315 (7)	−0.0067 (7)	−0.0050 (7)
S1	0.0419 (2)	0.0429 (2)	0.0323 (2)	−0.00887 (14)	−0.00199 (14)	0.00294 (13)

### Geometric parameters (Å, º)

**Table:**

C1—C5	1.383 (2)	C7—C8	1.3935 (19)
C1—C2	1.408 (2)	C7—C12	1.3974 (19)
C1—S1	1.7224 (14)	C8—C9	1.383 (2)
C2—N1	1.3400 (19)	C8—H8	0.9300
C2—N2	1.3836 (19)	C9—C10	1.375 (2)
C3—N1	1.332 (2)	C9—N3	1.4696 (18)
C3—C4	1.391 (2)	C10—C11	1.385 (2)
C3—H3	0.9300	C10—H10	0.9300
C4—C5	1.379 (2)	C11—C12	1.380 (2)
C4—H4	0.9300	C11—H11	0.9300
C5—H5	0.9300	C12—H12	0.9300
C6—N2	1.2980 (18)	N3—O1	1.2190 (18)
C6—C7	1.4705 (19)	N3—O2	1.221 (2)
C6—S1	1.7548 (14)		
			
C5—C1—C2	120.20 (13)	C9—C8—C7	118.53 (13)
C5—C1—S1	130.10 (12)	C9—C8—H8	120.7
C2—C1—S1	109.69 (11)	C7—C8—H8	120.7
N1—C2—N2	121.61 (13)	C10—C9—C8	123.22 (13)
N1—C2—C1	123.16 (14)	C10—C9—N3	118.65 (13)
N2—C2—C1	115.23 (12)	C8—C9—N3	118.12 (13)
N1—C3—C4	124.99 (14)	C9—C10—C11	117.85 (13)
N1—C3—H3	117.5	C9—C10—H10	121.1
C4—C3—H3	117.5	C11—C10—H10	121.1
C5—C4—C3	119.56 (15)	C12—C11—C10	120.58 (14)
C5—C4—H4	120.2	C12—C11—H11	119.7
C3—C4—H4	120.2	C10—C11—H11	119.7
C4—C5—C1	116.54 (14)	C11—C12—C7	120.90 (13)
C4—C5—H5	121.7	C11—C12—H12	119.5
C1—C5—H5	121.7	C7—C12—H12	119.5
N2—C6—C7	123.28 (12)	C3—N1—C2	115.53 (14)
N2—C6—S1	116.30 (11)	C6—N2—C2	110.10 (12)
C7—C6—S1	120.37 (10)	O1—N3—O2	123.28 (14)
C8—C7—C12	118.91 (13)	O1—N3—C9	118.36 (13)
C8—C7—C6	121.31 (12)	O2—N3—C9	118.35 (13)
C12—C7—C6	119.76 (12)	C1—S1—C6	88.68 (7)
