# Supplementary figures and images for: Crystal structure of 2-(3-nitro­phen­yl)-1,3-thia­zolo[4,5-b]pyridine
Source: Acta Crystallogr E Crystallogr Commun. 2015 Oct 24;71(Pt 11):o877. doi: 10.1107/S2056989015019118 (PMC4645065; doi:10.1107/S2056989015019118)

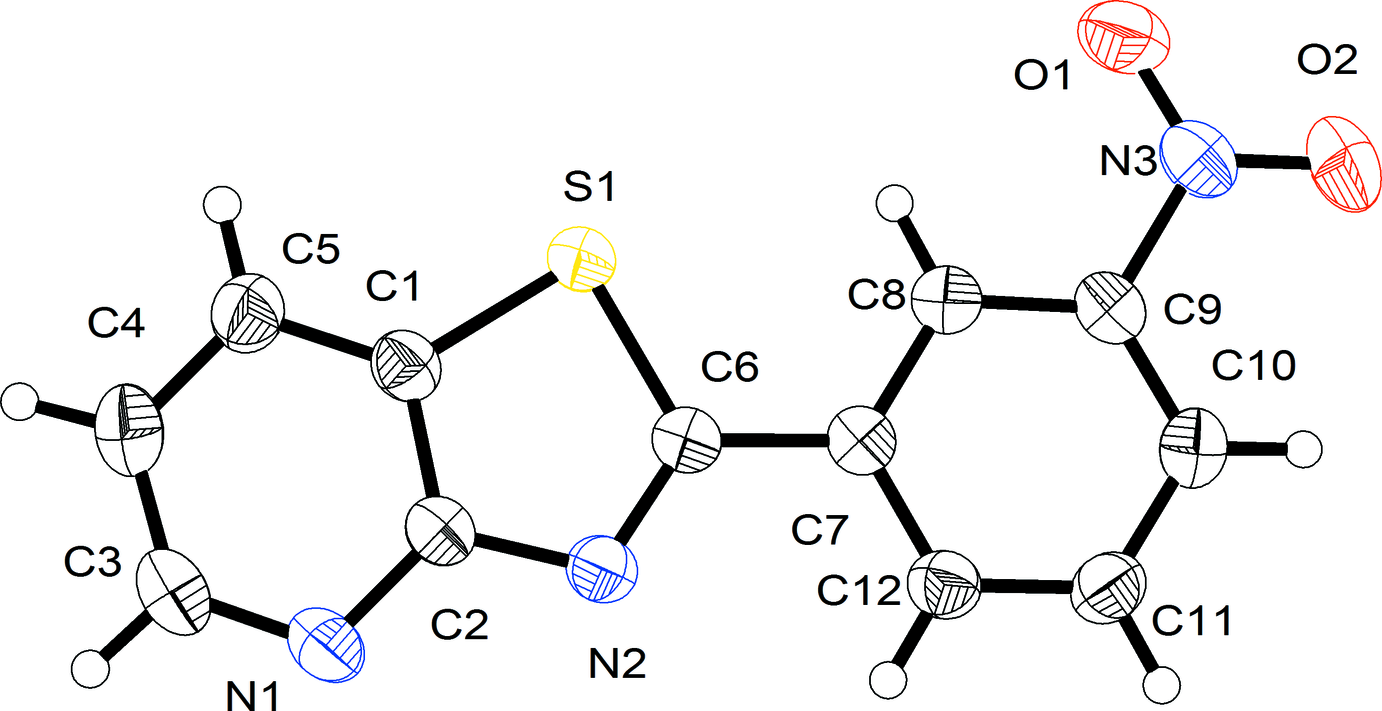

Supplement: Supplementary file 4 [file e-71-0o877-fig1.tif]

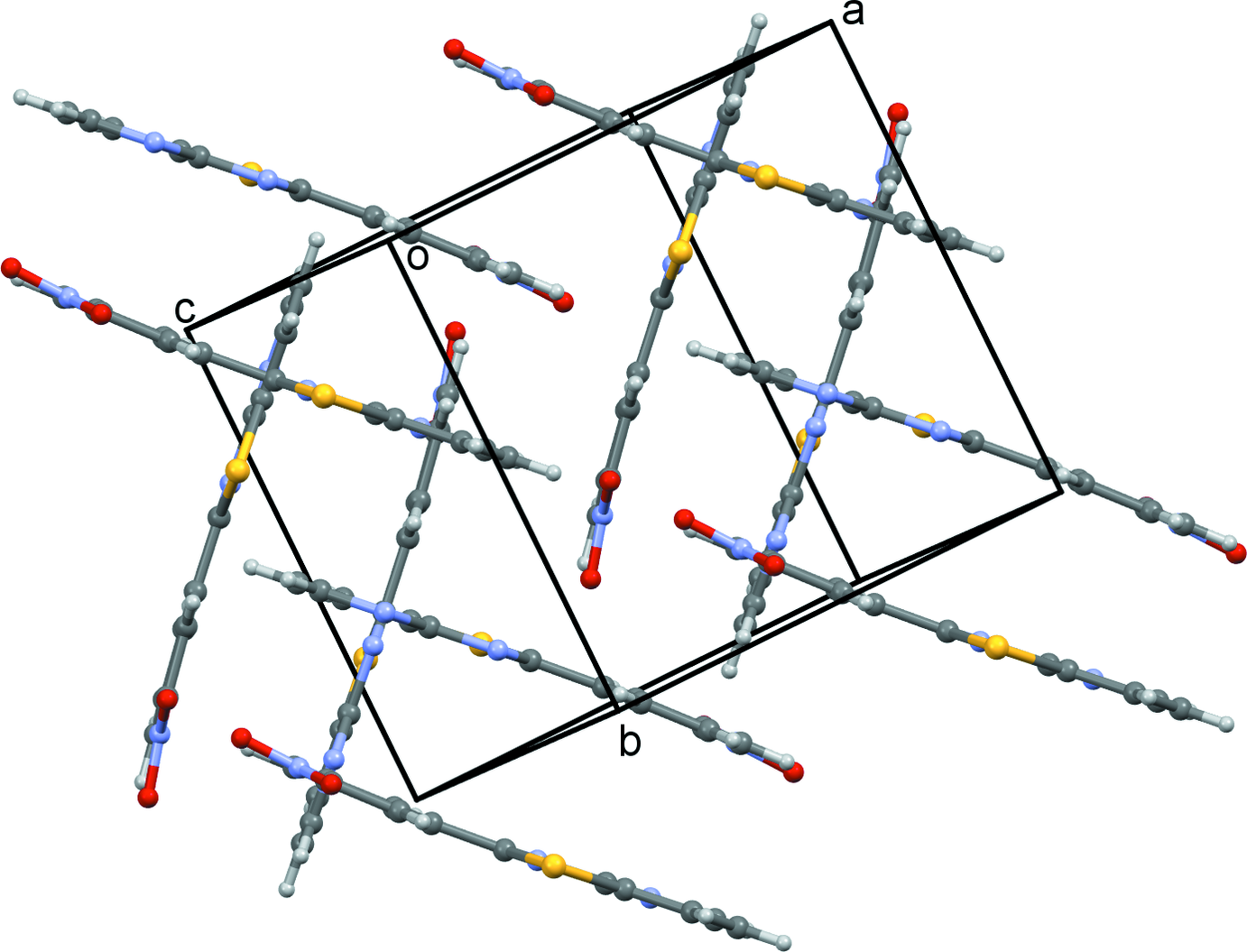

Supplement: Supplementary file 5 [file e-71-0o877-fig2.tif]
